# Patient preferences in the treatment of children with pollen-related allergic rhinitis

**DOI:** 10.1186/s13223-025-01004-y

**Published:** 2026-02-16

**Authors:** Ellen Tameeris-Kooiman, Arthur M. Bohnen, Patrick J. E. Bindels, Gijs Elshout

**Affiliations:** Department of General Practice, Erasmus Medical Center, Molewaterplein 40, 3015GD Rotterdam, The Netherlands

**Keywords:** Rhinitis, Allergic rhinitis, Patient preference, Pediatric, Adolescents, Shared decision making

## Abstract

**Background:**

Allergic rhinitis (AR) is a common condition affecting 23% of children in Europe. Of the AR-patients, 40–72% do not follow the treatment instructions as provided by their clinician. Shared decision making is effective in improving adherence to treatment. Knowing treatment preferences of children and adolescents can help improve shared decision making.

**Objective:**

To determine the preferential treatment of children and adolescents with AR.

**Methods:**

This study is a secondary analysis. Data from a single-blinded randomized controlled trial were used. The primary outcome measure of the original study was percentage of symptom-free days. Children aged 6–18 were randomised in three treatment groups: antihistamines (AH) on-demand, intranasal corticosteroids (INCS) continuous or INCS on-demand. Preferences were mapped out by questionnaires at the start and end of the study. The study design was approved by the Ethical Review Board of the Erasmus MC University Medical Center Rotterdam (No. MEC-2012-354). Prior to performing the trial, it was registered (Netherlands Trial Register No. NL3276).

**Results:**

At the end of the study AH on-demand was the preferential treatment arm (54.7%, *N* = 76/139). Preferences for continuous use over on-demand use and intranasal over oral therapy were dependent of treatment group (*p* = 0.027, *p* = 0.004). Of the participants, 36.7% chose their study medication as preferential treatment. Adolescents had more distinct treatment preferences than children (*p* = 0.004). Of the adolescents, 63% preferred AH on demand (*P* = 0.005), whereas children did not show a significant preference.

**Conclusion:**

There is a significant difference in preferences for treatment for AR between children and adolescents. AH on-demand is the most preferred medication type for AR. Shared decision making in choosing AR treatment should be used to improve the treatment compliance.

**Supplementary Information:**

The online version contains supplementary material available at 10.1186/s13223-025-01004-y.

## Introduction

Allergic rhinitis (AR) is a common disorder in children and adolescents with a prevalence of 23% in Europe [1]. The European Allergic Rhinitis and its Impact on Asthma (ARIA) and Dutch general practice guideline recommend oral use of antihistamines (AH), intranasal corticosteroids (INCS), intranasal AH or a combination of INCS and intranasal AH as first line treatment for intermittent rhinitis [2]. In the case of persistent rhinitis, a switch to another INCS and/or intranasal AH is recommended [3]. Baiardini performed a non-systematic literature review on adherence to medication among patients with AR. Overall, 40 to 72% of the AR patients follow the treatment instructions as provided by their clinician [4]. These numbers are based on a cross-sectional study among 349 children in Colombia (adherence 40%) and self-reported adherence data among Turkish children in a study evaluating reasons for non-adherence (adherence 72%) [5, 6]. Adherence levels to immunotherapy, despite large variation among studies, are higher (69–78%) [4]. Reasons for nonadherence are multifactorial. Side effects, high number of daily doses, a complex treatment plan and costs are factors that decrease adherence to treatment [4]. INCS are frequently discontinued due to the sensory aspects. Patients report nuisance from scent, taste and postnasal leakage of fluticasone propionate nasal spray (FPNS) [7]. Socio-economic status, gender, practical difficulties and additional environmental factors such as the number of children dependent of the caregiver, working hours, age of the children and multidrug use of the caregiver also affect adherence to medication [4, 5]. In adolescents, knowledge about the disease, beliefs about and attitude towards their disease, personal characteristics and communication are important factors that influence adherence [8]. 

Anti-allergic medication can be prescribed to be used continuously (on a daily basis) or on-demand (patients take medication only on days when they experience AR symptoms). In an observational study investigating the preference of physicians (*n* = 1008) in Spain, combination therapy (AH + INCS) was preferred and prescribed by 66%. Continuous use of medication was prescribed more often than on-demand use (58% continuous use in AH, 71% in INCS). In the same study, participants preferred oral treatment in 41% of the cases, 22% preferred intranasal therapy. Preference for combination treatment was not assessed [9]. Thanks to the rapid symptom relief, AH are suggested as first line therapy over INCS [10]. Other treatment options could then be added in case of insufficient symptom relief [11]. 

Shared decision making (SDM) has been found effective in improving adherence to treatment in other diseases than AR [12]. The recent ARIA guideline recommends caregivers to discuss the proposed treatment strategy with their patients [2]. To enable SDM, knowledge about patient preferences is needed, as SDM is an interactive discussion based on risks, benefits and patient preferences [12]. In addition, as AH are available over the counter (OTC), patients usually self-manage their disease, especially at onset or with mild AR. However, this can lead to suboptimal use of medication or therapy failure [13]. Knowledge about preferences can improve therapy adherence once they receive supervision in their therapy [14]. 

Patient preference studies have mainly focused on preferences between specific types of intranasal therapy for AR. For instance, the sensory aspects of fluticasone furoate nasal spray are preferred over other INCS such as mometasone furoate nasal spray and FPNS [7, 15–18]. Less is known about the patient preference between oral and intranasal treatment. Our aim is to assess which treatment method is preferred: intranasal versus oral and on-demand versus continuous in children with AR. These preferences are important since our randomized trial did not show large differences in symptom-free days between treatment groups [19]. 

## Methods

This study is part of a randomised controlled, single-blinded trial which focuses on symptomatic treatment of AR in children with mild-to-moderate AR caused by grass and birch pollen allergy. A detailed description of the study has been previously published [19]. The study design was approved by the Ethical Review Board of the Erasmus MC University Medical Center Rotterdam (No. MEC-2012-354). Prior to performing the trial, it was registered (Netherlands Trial Register No. NL3276).

### Patients and intervention

We recruited children aged 6–18 years old with pollen-related intermittent AR from general practitioners in The Netherlands. Patients were eligible for inclusion if they had an International Classification of Primary Care code (ICPC code) for AR or a previous prescription for AR medication (AH, INCS, or ocular antihistamines) registered in their electronic medical file in the year prior to inclusion. Participants needed a minimum retrospective symptom score of seven (out of maximum 21, appendix I) over the previous pollen season and sensitisation to grass pollen (determined by Fluorescent Enzyme Immunoassay [FEIA] class ≥ 2 or skin prick test wheal ≥ 3 mm). Patients were contacted by participating general practices in Rotterdam and surrounding towns and included after informed consent.

Included participants were randomly assigned to the treatment arms. The study period was carried out during the pollen season, from April 1st to July 31st (2013 & 2014). During this period, pollen count was reported. Birch pollen season lasted from April 23rd to May 13th (2013) and April 1st to April 23rd (2014); the grass pollen season lasted from May 23rd to July 31st (2013) and May 15th to June 28th (2014) [20]. Participants received either (1) INCS daily (FPNS, < 12 years old 100 mcg/day ≥ 12 years old 200 mcg/day), (2) INCS on-demand (FPNS, dosage as described in INCS continuous), or (3) AH on-demand (Levocetirizine dihydrochloride (diHCl) tablet, 5 mg/day). A treatment arm with continuous use of AH was not included, because the Dutch General Practice treatment standard does not recommend continuous treatment with AH [10]. Adherence to study medication was measured by counting returned medication (counting tablets for AH and weighing returned nasal spray for INCS). For INCS, the weight of one dose was measured beforehand to calculate the number of doses taken throughout the study period [19]. 

### Measurement of patient preferences

There was no validated patient preference questionnaire available for the paediatric study population. Patient preference was therefore measured by a questionnaire constructed for this study (appendix II). Questions were derived from the Treatment Satisfaction Questionnaire for Medication (TSQM), which is only validated for adults with, among other diseases, asthma [21]. The TSQM focuses on measuring patient satisfaction through evaluation of effectiveness, side effects, convenience of use and global satisfaction. Two questions of the TSQM were adapted for the paediatric study population (question 5 and 9). Differences in preference for oral versus intranasal medication were measured using questions derived from a study by Weinberg et al. on treatment preferences of adolescents with asthma [18]. The questions used were: ‘which was easier to use, medication A or medication B?’ and ‘Which did you prefer, taking medication A or medication B?’.

The questionnaire was administered face-to-face by a research assistant at baseline and after three months at endpoint. The baseline questionnaire consisted of 10 questions, which focused on what type of medication participants used in the previous season, when medication was used, how satisfied participants were with using the medication and which administration route they would prefer. Satisfaction was measured on a five-point scale (very annoying, annoying, neutral, pleasant and very pleasant). The endpoint questionnaire consisted of 13 questions. These focused on the satisfaction of participants with the use of the study medication, satisfaction with the symptom relief, the onset of action of the medication, the user-friendliness of the medication and overall satisfaction with the study medication. In addition, participants were asked to fill in their preferential administration route and frequency. Participants in the on-demand group were asked whether they found it difficult to estimate when they needed medication. Questionnaires were filled in by the children/participants themselves, unless they were not able to. In that case, parents/caregivers filled in the questionnaires.

At baseline, participants were asked to score the importance of different medication characteristics. We investigated whether participants found it important that medication had a rapid onset of action, was easy to take, had little side-effects and whether it was easy to see how much of the medication was left.

### Missing data

Because one of the aims of this study was to be able to evaluate change in preference between the baseline and endpoint of the study, only cases that filled in both the baseline and endpoint questionnaires were included in the analyses. There were no incomplete questionnaires.

### Statistical analysis

The five-point scale used in the questions about medication characteristics was dichotomised into two categories: a neutral to very positive opinion about the treatment and a negative to very negative opinion.

The primary outcome was patient preference after the treatment period, which was formulated in the endpoint-questionnaire as what type of treatment participants preferred (INCS daily, INCS on-demand or AH on-demand). To assure patient preferences were interpreted correctly, two comparisons were made in this trial: preference of daily use versus on-demand use of medication and intranasal (INCS) versus oral (AH) treatment, using a chi-square test. In addition, an explorative analysis on which factors influenced patient preferences was made using a multivariable logistic regression model. The use of medication in previous hay fever seasons, age, gender, treatment group, preference at the start of the study period, satisfaction about the use of the study medication and the opinion of participants about the study medication were initially included in the model, based on previous studies [17, 22]. The multivariable logistic regression model was built using backward selection. Variables were included in the model if the * p*-value was < 0.15. Confounders were identified when they elicited a change of >15% in the remaining parameter estimates [23]. The fit of the model was assessed using he Nagelkerke R^2^ and the Hosmer-Lemeshow goodness-of-fit test.

Data were analysed using IBM SPSS Statistics version 28 for Windows (SPSS Inc., Chigago, IL).

## Results

### Patient characteristics

**Fig. 1:**
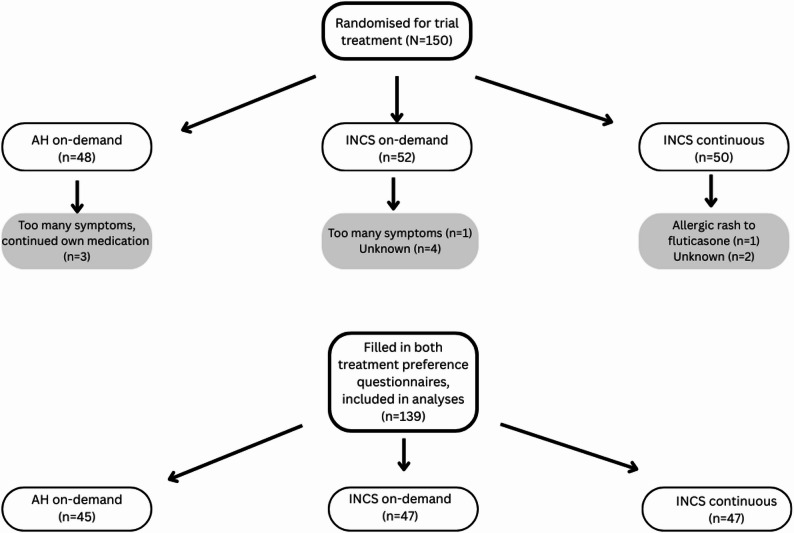
Summary of patient selection

150 participants were randomised to AH on-demand (*N* = 48), INCS continuous (*N* = 50) and to INCS on-demand (*N* = 52). In total, 139 participants filled in both the baseline and endpoint patient preference questionnaire and were included for further analyses (Fig. 1).

Baseline characteristics of the study population are shown in Table 1. Mean age of all participants was 11.6 years (SD 3.3), 51.4% of the participants were male, 70.5% of the participants were adolescents (≥ 10 years). There were no significant differences in baseline characteristics between the treatment groups. Adherence to medication during the study period was good (>75%) to moderate [4]: children in the INCS continuous group took 88% of the prescribed doses, adolescents 73%. In the INCS on-demand group, children took 33% of the maximum doses, adolescents 22%. In the AH on-demand group, children and adolescents took 37% and 42% of the maximum prescribed doses (Table 2).

**Table 1:** Baseline characteristics of the study population

	AH on-demand N = 45	INCS continuous N = 47	INCS on-demand N = 47
Sex (male, N (%))	23 (51.1)	20 (42.6)	26 (55.3)
Age (mean (SD))	11.22 (3.31)	12.24 (3.34)	11.77 (3.03)
Adolescents (%)	29 (64.4)	36 (76.6)	35 (74.5)
Mean symptom score in previous season (SD)	12.36 (3.76)	11.64 (3.11)	12.21 (2.98)
Birch pollen sensitisation (%)	25 (55.6)	29 (61.7)	27 (57.4)
HDM sensitisation (%)	20 (44.4)	24 (51.1)	27 (57.4)
Asthma (%)	9 (20.0)	14 (29.8)	6 (12.8)

**Table 2:** Adherence to medication by randomisation group

	AH on-demand	INCS continuous	INCS on-demand
Children	37 (25–49)	88 (63–114)	33 (18–48)
Adolescents	42 (29–54)	73 (61–86)	22 (15–30)

% of max. prescribed doses taken (95% confidence interval)

### Loss to follow-up

Of the 139 participants in the current analysis, seven did not adhere to the treatment protocol. Of these participants, three had too many or severe symptoms and re-started their own pre-study medication (all in the AH on-demand group), one had an allergic rash due to the fluticasone (INCS continuous group). For the other three, the reason for dropout is not known. As all of these participants did fill in the preference questionnaire at the end of the study period, they have been included in the analysis (Figure I).

### AR treatment in previous season

Of the total study population (*n* = 150), 100 (66.7%) participants filled in the questions about satisfaction about previous medication. Of the participants, 70 (46.6%) used a combination of tablets and intranasal spray in the previous season, 21 (14.0%) used tablets only and 16 (10.6%) used intranasal spray. There were no significant differences in previously used medication between the treatment groups. Of the participants, 58% (*n* = 87) had been using their medication daily in the previous season and 31.3% (*n* = 47) of the participants valued their previously used medication as (very) pleasant to use.

### Importance of medication characteristics

**Fig. 2:**
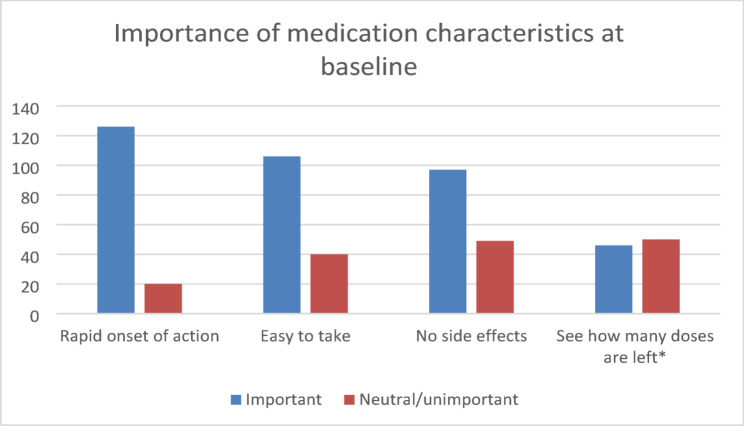
Importance of medication characteristics at baseline. * this question was filled in by 96 participants (34% missing)

When asked what type of application participants favoured for their AR treatment at baseline, 14.4% (*n* = 21) preferred tablets and 11.0% (*N* = 16) preferred a nasal spray. Combined treatment of tablets and nasal spray was preferred by 39 participants (26.7%), 64 (43.8%) preferred (combined) ocular therapy, 4.1% (*N* = 6) had no preference for administration route. Of the participants, 86.3% (*n* = 126) found it important that medication had a rapid onset of action and 72.6% (*n* = 106) found user friendliness important (Fig. 2).

### Patient satisfaction at the end of the study period

Of the participants, 126 (90.6%) were satisfied with their study medication at the end of the study period (rated their study medication as overall neutral, satisfied, or very satisfied). Symptom reduction of the study medication was rated as satisfactory by 83.5% (*N* = 116) of the participants, 84.2% (*N* = 117) of the study participants was satisfied with the time between onset of symptoms and symptom relief by the used study medication.

### Patient medication preferences over time

At the start of the study period, 57.6% (*N* = 80) of the participants preferred AH on-demand as therapy for their AR, 17.3% (*N* = 24) favoured INCS continuous, 25.2% (*N* = 35) preferred INCS on-demand (Table 3). There were no significant differences in preference among the treatment groups (Pearson Chi-Square 1.83, *p* = 0.767).

**Table 3:** Importance of medication characteristics at baseline

Characteristic		N	%
Rapid onset of action	(very) important	126	86
	Neutral/ unimportant	20	14
Easy to take	(very) important	106	73
	Neutral/ unimportant	40	27
No side effects	(very) important	97	66
	Neutral/ unimportant	49	34
See how many doses are left*	(very) important	46	48
	Neutral/ unimportant	50	52

* this question was filled in by 96 participants (34% missing)

We observed that 59.6% (*n* = 87) of the participants did not change their preference for medication over the study period. There were no significant differences between the treatment groups when it comes to change of preference from baseline to endpoint (Pearson Chi-Square 0.372, *p* = 0.830). At the end of the study period, 54.7% (*N* = 76) of the participants preferred AH on-demand as treatment for their AR symptoms, 23.0% (*N* = 32) preferred INCS on-demand and 14.4% (*N* = 20) preferred continuous use of INCS (Table 3). Of the participants 7.9% had no preferential treatment. AH was not selected as preferential medication by participants in the INCS continuous and INCS on-demand groups. They mostly chose the medication group in which they were randomised as preferential treatment method (*p* = 0.013, Table 3). In total, 36.7% of the participants (*n* = 51) chose the treatment arm they were randomised to as their preferential treatment method. Participants that were randomised to their preferential treatment method (*N* = 52), did not change their preferential treatment method in 26.9% (*N* = 14).

### Patient preference for dosage and administration route

On-demand use was preferred over continuous use by 84.4% of the participants. The preference was dependent of treatment group ( *p* = 0.027). Of the participants, 59.4% preferred oral therapy over intranasal therapy (Table 4).

**Table 4:** Patient preferences

		Randomisation			Total
		AH on-demand	INCS continuous	INCS on-demand	
Preference (start of study period)	AH on-demand	29 (64.4)	24 (51.1)	27 (57.4)	80 (57.6)†
	INCS continuous	6 (13.3)	10 (21.3)	8 (17.0)	24 (17.3)†
	INCS on-demand	10 (22.2)	13 (27.7)	12 (25.5)	35 (25.2)†
Total		45 (100)	47 (100)	47 (100)	139
		AH on-demand	INCS continuous	INCS on-demand	Total
Preference (end of study period)	AH on-demand	33 (73.3)	21 (44.7)	22 (46.8)	76 (54.7)†
	INCS continuous	3 (6.7)	12 (25.5)	5 (10.6)	20 (14.4)†
	INCS on-demand	5 (11.1)	11 (23.4)	16 (34.0)	32 (23.0)†
	No preference	4 (8.9)	3 (6.4)	4 (8.5)	11 (7.9)†
Total		45 (100)	47 (100)	47 (100)	139
		AH on-demand	INCS continuous	INCS on-demand	Total
Preference (administra-tion route)	On-demand	38 (92.7)	32 (72.7)	38 (88.4)	108 (84.4)
	Continuous	3 (7.3)	12 (27.3)	5 (11.6)	20 (15.6)
	Tablets	33 (80.5)	21 (47.7)	22 (51.2)	76 (59.4)
	Nasal spray	8 (19.5)	23 (52.3)	21 (48.8)	52 (40.6)

N (% within randomisation group); † % within total study population

### Differences between children and adolescents

There was a significant difference in preference between children and adolescents (*p* = 0.004). Adolescents favoured AH on demand (63.0%, subanalysis among adolescents *p* = 0.005), whereas the preference of children was equally spread over the three treatment groups (sub analysis among children: *p* = 0.502) (Table 5). Of the children in the study population, 51.3% (*n* = 20) preferred intranasal therapy over tablets (33.3%, *n* = 13), 15.4% (*n* = 6) of the children’s responses were missing. Of the children, 59% (*n* = 23) preferred on-demand therapy over continuous use of medication, 10 children (25.6%) preferred continuous use. Of the adolescents, 63.0% (*n* = 63) preferred oral therapy over intranasal spray (32.0%, *n* = 32). The preference of 5 (5.0%) adolescents was missing. There was a significant difference in preference between the treatment groups (*p* = 0.008). Of the adolescents, 85% preferred on-demand prescription of their medication, versus 10.0% that preferred continuous use. Of the adolescents preferring continuous use of medication, the biggest proportion (72.7%) was randomised to continuous use of INCS (*p* = 0.011). The difference in preference for oral or intranasal therapy between children and adolescents was significant (*p* = 0.007). The difference in preference for continuous or on-demand therapy was also significant (*p* = 0.007). (Table 5).

**Table 5:** Preferences per age group

		Children	Adolescents	Total
Preference	AH on-demand	13 (33.3)	63 (63.0)	76 (54.7)
	INCS continuous	10 (25.6)	10 (10.0)	20 (14.4)
	INCS on-demand	10 (25.6)	22 (22.0)	32 (23.0)
	No preference	6 (15.4)	5 (5.0)	11 (7.9)
	TOTAL	39 (100)	100 (100)	139

N (% within age group)

### Multivariate logistic regression

Preference for both continuous vs. on-demand use and oral vs. intranasal use of medication was dependent of treatment group. Adolescent age contributed to the preference for continuous vs. on-demand use. Sex and preference at the start of the study period were associated with preference for oral vs. intranasal use of medication (Appendix III).

## Discussion

The participants in our study preferred on-demand use of medication over continuous use (84.4% vs. 15.6%, respectively). There was a less distinctive difference in preference for tablets (59.4%) over intranasal spray (40.6%) in the complete study group. However, when focusing on the adolescents in the study population, there was a significant preference for tablets (63.0%). Overall, the participants preferred AH on-demand as therapy for their AR over continuous use of INCS and INCS on-demand. The majority of the participants (59.9%) did not change their preference throughout the study period.

A limitation of this secondary analysis is the design of the trial, a cross-over design might have been preferable to study preferences. However, since most of the patients experienced various treatments either in the past or in the present study we believe that they are able to make a proper judgement about their own preferences. Our results (about influence of previous treatments, about differences between children and adolescents, and about AH being the preferred treatment) are valuable for daily practice. We expect a crossover design to reveal similar results.

In a study among adults from Sevilla, Spain with AR by Navarro et al. (*n* = 4040, mean age 34 years), 41% of the participants preferred an oral administration route of their medication, versus 22% that preferred intranasal therapy [9]. Wong et al. investigated treatment preferences of children in Singapore with AR and experience with topical nasal treatment (*n* = 400, mean age: 7.54 years) and found that 73% of the participants preferred oral over intranasal (11%) administration of medication [24]. These findings are in line with the results found in this study.

The sensory aspects of intranasal corticosteroids are one of the reasons for patients to discontinue their treatment [7, 24]. This could explain the preference of the participants in our study to AH over INCS. The participants in our study were not asked about the sensory experience of the study medication. However, fluticasone propionate, the INCS used in this randomized controlled trial (RCT), has been shown to have sensory components that patients find unfavorable compared to budesonide or mometasone [25, 26]. Of the participants that labeled their study medication as unpleasant to use (*N* = 13), 10 used INCS (continuous or on-demand). This finding supports the hypothesis that the sensory aspect of the INCS might have influenced the outcomes. Since the patient satisfaction and preference questionnaires were only handed out at the end of the study period, recall bias might have occurred.

The majority (72.7%) of the participants randomised to continuous use of INCS preferred on-demand use of their medication. The participants in the INCS continuous group, were adherent to their medication in 88% (children) and 73% (adolescents) of the days in the study period. There are not many studies describing adherence in AR, but the numbers reported in literature are similar to the adherence we found in our study [4]. The results found in this study can therefore be applied to the general population of children and adolescents being prescribed continuous use of INCS.

Children randomised into the on-demand treatment arms had a preference for on-demand treatment more often than those randomised to use their medication continuously (88.4% vs. 72.7%, respectively). Treatment group contributed significantly to predict patient preference in the logistic regression models. Even though the amounts of study participants in each group of the models are small, this might indicate that patients prefer treatment options that they are familiar with. However, the preferences could also be biased by exposure to the intervention. A crossover study design might give better insight in future research.

Even though the majority of the participants did not change their preference over the study period, a substantial 39.1% of the participants did change their preference over time. The reasons for the change of preference did not become clear in our study; this leaves potential for future research. These results should be taken into account in clinical practice. When evaluating AR treatment, clinicians should take into account that preferences of patients might change over time, possibly due to treatment satisfaction.

The preference of participants for a combination therapy is not taken into account in this study since participants were not able to select the combination as their preferred AR treatment. In the season prior to the study, 47.9% of the participants used a combination of nasal spray and tablets for their AR. These numbers are in line with previously reported prescription habits of physicians [27]. The preferences of participants might have been different if it would have been possible for participants to select a combination of therapies as a preferential method.

In a previous analysis of the current dataset, the treatment options have been found to be non-inferior to each other when it comes to percentage of symptom-free days [19]. The most recent ARIA guideline indicates AH and INCS as equal treatment options in the first line [3]. The preference of patients and the importance of shared decision making are also emphasized in recent guidelines [28]. AH on-demand is the preferential treatment option in our study.

Therapy adherence can be improved by using the preferential treatment method of patients [29]. Therefore, it is important to take patient preferences into account when setting up a treatment plan. This study makes clear that there are significant differences in treatment preferences between children and adolescents and that previously used medication contributes to the preference of the patient. As the original trial showed non-inferiority of the treatment arms in efficacy and the present analysis shows that a vast majority of patients express a clear preference, in many cases based on previous experiences, these preferences can contribute to substantiated treatment decisions. If patients are to start their first medication for AR symptoms, the results of this study can help provide an evidence based approach on the preferences of children and adolescents in AR treatment.

## Supplementary Information

Below is the link to the electronic supplementary material.

Supplementary Material 1.

## Data Availability

Data can be provided upon request.

## Appendix I–screening questionnaire

This questionnaire was sent to participants meeting inclusion criteria in their patient file. Eligibility was further investigated using the symptom score for the past pollen season. A minimum of 7 out of 21 points was required. The original questionnaire was presented in Dutch.

1. Does your child have hay fever (grass or tree pollen allergy)? *Answer options: yes/no/unknown.*
2. Did your child experience hay fever symptoms during last hay fever season (March–August)? *Answer options: yes/no/unknown.*
3. Did your child experience one of the following complaints during last hay fever season (March–August)?
  - Sneezing.
  - Itching nose.
  - Runny nose.
  - Nasal congestion.
  - Teary eyes.
  - Itching eyes.
  - Red eyes.

*Answer options: 0–none*, *1–little*, *2–much*, *3–very much*.

Original questionnaire:

## Appendix II–patient preference questionnaires

These questionnaires were originally performed in Dutch.

**Patient medication preference questionnaire (start of study)**

1. Which type of medication did you use during last hay fever season?
  - None.
  - Tablets.
  - Nasal spray.
  - Nasal drops.
  - Ocular drops.
  - Other ….
  - I can’t remember.
2. When did you use this medication?
  - The whole hay fever season.
  - Only when I had symptoms.
  - I don’t know.
  - Other ….
3. How often did you use the medication during the hay fever season?
  - Daily.
  - (at least) 3x per week.
  - (at least) 1x per week.
  - (at least) every other week.
  - (at least) once a month.
  - Less than once a month.
4. How did you experience using this medication?
  - Very pleasant.
  - Pleasant.
  - Neutral.
  - Unpleasant.
  - Very unpleasant.
5. Which form of medication would you like to use for your allergy symptoms?
  - Tablets.
  - Nasal spray.
  - Nasal drops.
  - Ocular drops.
  - Other….
  - No preference.
  - I don’t know.
6. In this study you will be randomized to one of the three treatment groups. Which treatment has your preference?
  - Tablets only when hay fever symptoms arise.
  - Nasal spray only when hay fever symptoms arise.
  - Nasal spray during the whole hay fever season (daily).
7. How important are the following aspects when using your allergy medication?
  - Medication works quickly.
    i. Very important.
    ii. Important.
    iii. Neutral.
    iv. Unimportant.
    v. Very unimportant.
  - Medication is easy to take.
    i. Very important.
    ii. Important.
    iii. Neutral.
    iv. Unimportant.
    v. Very unimportant.
  - Medication has few side effects.
    i. Very important.
    ii. Important.
    iii. Neutral.
    iv. Unimportant.
    v. Very unimportant.
  - You can see how much of your medication is left.
    i. Very important.
    ii. Important.
    iii. Neutral.
    iv. Unimportant.
    v. Very unimportant.

**Patient medication preference questionnaire (end of study)**

1. How did you experience using the study medication?
  - Very pleasant.
  - Pleasant.
  - Neutral.
  - Unpleasant.
  - Very unpleasant.
2. How satisfied are you with the ability of the medication to treat your hay fever?
  - Very satisfied.
  - Satisfied.
  - Neutral.
  - Unsatisfied.
  - Very satisfied.
3. How satisfied are you with the way the medication reliefs the hay fever symptoms?
  - Very satisfied.
  - Satisfied.
  - Neutral.
  - Unsatisfied.
  - Very satisfied.
4. How satisfied are you with the time it takes before onset of action of the medication?
  - Very satisfied.
  - Satisfied.
  - Neutral.
  - Unsatisfied.
  - Very satisfied.
5. How easy is it to take the medication?
  - Very easy.
  - Easy.
  - Neutral.
  - Difficult.
  - Very difficult.
6. How convenient is it to take the medication as instructed?
  - Very convenient.
  - Convenient.
  - Neutral.
  - Inconvenient.
  - Very inconvenient.
7. In total, how convinced are you that taking this medication is good for you?
  - Very convinced.
  - Convinced.
  - Neutral.
  - Unconvinced.
  - Very unconvinced.
8. How sure are you that the good things about this medication outweigh the bad?
  - Very sure.
  - Sure.
  - Neutral.
  - Not sure.
  - Very unsure.
9. In total, how satisfied are you with the medication?
  - Very satisfied.
  - Satisfied.
  - Neutral.
  - Unsatisfied.
  - Very satisfied.
10. In this study you were randomized to one of the three treatment arms. Imagine you would be able to choose your own medication, which one would you choose?
  - Tablets only when hay fever symptoms arise.
  - Nasal spray only when hay fever symptoms arise.
  - Nasal spray during the whole hay fever season (daily).
11. Which form of medication would you preferably use to alleviate hay fever symptoms?
  - None.
  - Tablets.
  - Nasal spray.
  - Nasal drops.
  - Ocular drops.
  - Other ….
  - No preference.
  - I don’t know.

## Appendix III–outcomes multivariate logistic regression

**Table:**

Variable	Exp(B)	*p*-value (Sig.)	95% C.I. for Exp(B)	Lower	Upper
Age	0.746	0.061	0.549–1.014	1.014	
Sex	0.150	0.005	0.040–0.559	0.559	
Adolescent	1.280	0.829	0.137–11.985	11.985	
Randomisation (AH, reference)		< 0.001			
Randomisation (INCS continuous)	22.062	< 0.001	4.506	108.015	
Randomisation (INCS on-demand)	6.341	0.021	1.323	30.397	
Previously used medication (intranasal spray, reference)		0.420			
Previously used medication (tablets)	0.926	0.937	0.136	6.326	
Previously used medication (tablets & spray)	0.717	0.665	0.159	3.240	
Previously used medication (other)	0.168	0.205	0.011	2.653	
Previously used medication (no medication)	0.053	0.093	0.002	1.640	
PVA_preference (AH, reference)		< 0.001			
PVA_preference (INCS continuous)	28.224	< 0.001	4.752	167.647	
PVA_preference (INCS on-demand)	15.171	0.001	2.957	77.839	
Satisfaction with study medication	0.254	0.152	0.039	1.660	
Constant	34.823	0.066			
