# Supplementary material for: Patient preferences in the treatment of children with pollen-related allergic rhinitis
Source: Allergy Asthma Clin Immunol. 2026 Feb 16;22:13. doi: 10.1186/s13223-025-01004-y (PMC13015076; doi:10.1186/s13223-025-01004-y)
Supplement: Supplementary file 1 — Supplementary Material 1. [file 13223_2025_1004_MOESM1_ESM.docx]

**eAppendix 1 – multivariable analysis for preference of participants**

eTable 1 - Multivariable analysis for preference for continuous use of medication

| Variable |  | OR | 95% CI |
| --- | --- | --- | --- |
| Adolescent |  | 0.142 | 0.011-0.179 |
| Randomisation | AH on-demand | Reference |  |
|  | INCS continuous | 10.926 | 1.612-74.048 |
|  | INCS on-demand | 1.756 | 0.234-13.191 |
| Preference at start of study period | Antihistamine | Reference |  |
|  | INCS continuous | 7.530 | 1.496-37.906 |
|  | INCS on-demand | 0.847 | 0.160-4.499 |

Overall fit of the model: moderate (Nagelkerke R-square 0.326, Hosmer Lemeshow p = 0.281)

eTable 2 - Multivariable analysis for preference for intranasal use of medication

| Variable |  | OR | 95% CI |
| --- | --- | --- | --- |
| Sex (male) |  | 0.199 | 0.058-0.679 |
| Randomisation | AH on-demand | Reference |  |
|  | INCS continous | 20.042 | 4.196-95.731 |
|  | INCS on-demand | 6.793 | 1.488-30.997 |
| Preference at start of the study period | Antihistamine | Reference |  |
|  | INCS continuous | 28.224 | 4.752-167.647 |
|  | INCS on-demand | 15.171 | 2.957-77.839 |

Overall fit of the model: good (Nagelkerke 0.579, Hosmer Lemeshow p = 0.473)

**eAppendix 2 – Screening questionnaire**

This questionnaire was sent to participants meeting inclusion criteria in their patient file. Eligibility was further investigated using the symptom score for the past pollen season. A minimum of 7 out of 21 points was required. The original questionnaire was presented in Dutch.

1. Does your child have hay fever (grass or tree pollen allergy)? *Answer options: yes/no/unknown.*
2. Did your child experience hay fever symptoms during last hay fever season (March – August)? *Answer options: yes/no/unknown.*
3. Did your child experience one of the following complaints during last hay fever season (March – August)?
   1. Sneezing
   2. Itching nose
   3. Runny nose
   4. Nasal congestion
   5. Teary eyes
   6. Itching eyes
   7. Red eyes

*Answer options: 0 – none, 1 – little, 2 – much, 3 – very much*

Original questionnaire:


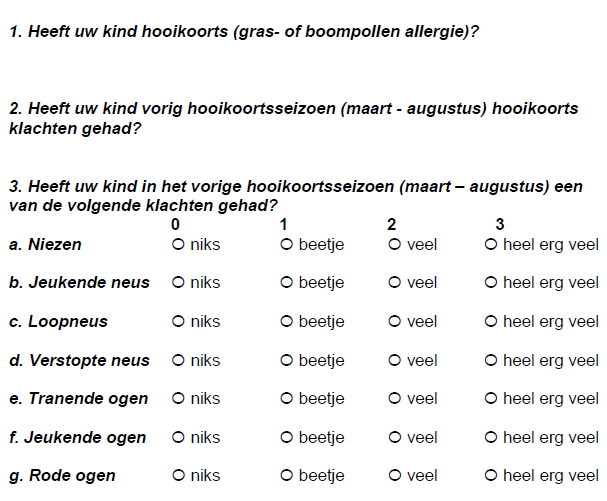


**eAppendix 3 – patient preference questionnaires**

These questionnaires were originally performed in Dutch

**Patient medication preference questionnaire (start of study)**

1. Which type of medication did you use during last hay fever season?
   1. None
   2. Tablets
   3. Nasal spray
   4. Nasal drops
   5. Ocular drops
   6. Other …
   7. I can’t remember
2. When did you use this medication?
   1. The whole hay fever season
   2. Only when I had symptoms
   3. I don’t know
   4. Other …
3. How often did you use the medication during the hay fever season?
   1. Daily
   2. (at least) 3x per week
   3. (at least) 1x per week
   4. (at least) every other week
   5. (at least) once a month
   6. Less than once a month
4. How did you experience using this medication?
   1. Very pleasant
   2. Pleasant
   3. Neutral
   4. Unpleasant
   5. Very unpleasant
5. Which form of medication would you like to use for your allergy symptoms?
   1. Tablets
   2. Nasal spray
   3. Nasal drops
   4. Ocular drops
   5. Other…
   6. No preference
   7. I don’t know
6. In this study you will be randomized to one of the three treatment groups. Which treatment has your preference?
   1. Tablets only when hay fever symptoms arise
   2. Nasal spray only when hay fever symptoms arise
   3. Nasal spray during the whole hay fever season (daily)
7. How important are the following aspects when using your allergy medication?
   1. Medication works quickly
      1. Very important
      2. Important
      3. Neutral
      4. Unimportant
      5. Very unimportant
   2. Medication is easy to take
      1. Very important
      2. Important
      3. Neutral
      4. Unimportant
      5. Very unimportant
   3. Medication has few side effects
      1. Very important
      2. Important
      3. Neutral
      4. Unimportant
      5. Very unimportant
   4. You can see how much of your medication is left
      1. Very important
      2. Important
      3. Neutral
      4. Unimportant
      5. Very unimportant

**Patient medication preference questionnaire (end of study)**

1. How did you experience using the study medication?
   1. Very pleasant
   2. Pleasant
   3. Neutral
   4. Unpleasant
   5. Very unpleasant
2. How satisfied are you with the ability of the medication to treat your hay fever?
   1. Very satisfied
   2. Satisfied
   3. Neutral
   4. Unsatisfied
   5. Very satisfied
3. How satisfied are you with the way the medication reliefs the hay fever symptoms?
   1. Very satisfied
   2. Satisfied
   3. Neutral
   4. Unsatisfied
   5. Very satisfied
4. How satisfied are you with the time it takes before onset of action of the medication?
   1. Very satisfied
   2. Satisfied
   3. Neutral
   4. Unsatisfied
   5. Very satisfied
5. How easy is it to take the medication?
   1. Very easy
   2. Easy
   3. Neutral
   4. Difficult
   5. Very difficult
6. How convenient is it to take the medication as instructed?
   1. Very convenient
   2. Convenient
   3. Neutral
   4. Inconvenient
   5. Very inconvenient
7. In total, how convinced are you that taking this medication is good for you?
   1. Very convinced
   2. Convinced
   3. Neutral
   4. Unconvinced
   5. Very unconvinced
8. How sure are you that the good things about this medication outweigh the bad?
   1. Very sure
   2. Sure
   3. Neutral
   4. Not sure
   5. Very unsure
9. In total, how satisfied are you with the medication?
   1. Very satisfied
   2. Satisfied
   3. Neutral
   4. Unsatisfied
   5. Very satisfied
10. In this study you were randomized to one of the three treatment arms. Imagine you would be able to choose your own medication, which one would you choose?
    1. Tablets only when hay fever symptoms arise
    2. Nasal spray only when hay fever symptoms arise
    3. Nasal spray during the whole hay fever season (daily)
11. Which form of medication would you preferably use to alleviate hay fever symptoms?
    1. None
    2. Tablets
    3. Nasal spray
    4. Nasal drops
    5. Ocular drops
    6. Other …
    7. No preference
    8. I don’t know
